# High Risk of Anxiety and Depression in Women With Different Types of Pregnancy Complications in France: A Cross-Sectional Study

**DOI:** 10.1155/jp/9221290

**Published:** 2025-12-01

**Authors:** Jaqueline Wendland, Camila Teixeira Ribeiro, Mélina Audic, Jessica Letot, Shukrullah Ahmadi

**Affiliations:** Psychopathology and Health Processes Laboratory, University Paris Cité, Boulogne Billancourt, France

**Keywords:** anxiety, complications, depression, pregnancy

## Abstract

**Background:**

Pregnancy complications are known to be risk factors for the onset of depression and anxiety symptoms. This study assessed associations between pregnancy complications, including concurrent complications, and symptoms of anxiety and depression among pregnant women living in France.

**Methods:**

A cross-sectional study was carried out among 492 pregnant women. Sociodemographic and obstetric characteristics were collected using an online questionnaire. Depression and anxiety symptoms were evaluated using the Edinburgh Postnatal Depression Scale and the Spielberger State–Trait Anxiety Inventory, respectively. Multivariate logistic regressions were employed to identify associations between mental health outcomes and pregnancy complications.

**Results:**

While 37% of women declared no pregnancy complications, 9.76% declared two or more complications, and 63% of participants had at least one complication. Among these latter, 68.9% had a high risk of depression, 83.9% elevated state anxiety, and 77.4% elevated trait anxiety. State anxiety scores were significantly higher in women who felt they did not receive adequate social support from their partner, family, and friends and who reported dissatisfaction with medical care. Adjusting for confounders, we identified that women with complications had higher odds of experiencing higher state anxiety scores (adjusted OR: 2.94; 95% CI: 1.40–6.10). Positive associations were also observed between gestational diabetes mellitus and increased likelihood of reporting depressive symptoms (adjusted OR: 1.99; CI:1. 20–3.29) and high state anxiety scores (OR: 3.31; CI: 1.22–9.01).

**Conclusion:**

We found a high prevalence of depression and anxiety among pregnant women with complications. Gestational diabetes mellitus was positively associated with antenatal depression and high state anxiety levels. These findings suggest that women with complications have a higher risk of developing depressive and anxious symptoms. Screening for and treating physical and mental health problems in women experiencing pregnancy complications and poor mental health symptoms are crucial to safeguard the well-being of the mother and the fetus.

## 1. Introduction

Pregnancy is a major and unique moment in a woman's life, often perceived as a time of joy and anticipation of a happy event. Expecting a child is also a transitional state that engenders substantial physical, physiological, and psychological changes, which may lead to the onset of antenatal and childbirth complications. Over 20% of pregnant women develop one or more medical complications that pose a threat to the well-being of the mother or fetus, or both [1]. The gestational period can trigger symptoms of depression and anxiety [2], as lifestyle changes and management of high-risk pregnancy impact pregnant women's coping mechanisms and their well-being, potentially increasing their risk for psychopathological conditions [3]. Approximately 12.5% of women develop an antenatal mental health problem in France according to findings of ELFE cohort [4].

In France, every year, one in five women experiences complications during pregnancy that can jeopardize maternal or child health outcomes [5]. Among complications, the risk of preterm delivery is the most frequent (7%) and one of the leading causes of antenatal hospitalization, affecting 4.8% of pregnancies in France [5]. It refers to the risk that the birth of a baby occurs before 37 weeks due to medical or obstetric factors. It is characterized by the appearance of spontaneous uterine contractions and a change in the cervix. It is associated with an increased risk of poor maternal and neonatal outcomes. Gestational diabetes mellitus (GDM), hypertensive disorders (4.3%), and placenta previa (1.5%) are also very common gestational conditions. GDM, characterized as a type of diabetes mellitus that has its onset after the first trimester of pregnancy [6, 7], was 14% prevalent among pregnant women globally in 2021, with estimations of 7.8% in Europe [8]. The incidence of GDM is rising in France, with 16.4% of women diagnosed with it in 2021 versus 10.9% in 2016 [6]. Despite its high occurrence, there is limited evidence of the association between GDM and antenatal anxiety [9], and a review on this subject has evaluated associations between GDM and postpartum depression only [10].

The occurrence of pregnancy complications, which inevitably generate a certain degree of stress, can be a risk factor for anxiety and depressive symptoms in pregnant women [11]. Exposure to certain additional risk factors, such as poor socioeconomic factors or low social support from the partner or family, can exacerbate these symptoms [12]. Current evidence from a systematic review suggests that one in three women experiencing pregnancy complications report considerable levels of depression or anxiety [13]. The prevalence of antepartum anxiety and depression among women hospitalized with obstetric complications is estimated to be around 29% and 34%, respectively [13].

Recent reviews suggest that most research on the prevalence and risk of perinatal mental health problems among women with pregnancy complications has been mainly conducted outside France [2, 9, 13, 14]. Similarly, most of the available studies have focused on a single type of complication (e.g., DGM) without considering the separate or combined impact of different types of complications. Therefore, the objective of this study is to examine associations between different types of pregnancy complications (single or multiple) and symptoms of anxiety and depression.

## 2. Materials and Methods

### 2.1. Population

This is a cross-sectional study conducted in France in 2019–2020, before the Covid-19 pandemic, among 492 pregnant women. Pregnant women with or without one or more complications of pregnancy were invited to take part in an online study. The inclusion criteria were to be an adult woman in the course of pregnancy and to be fluent in French (read and written). The exclusion criteria were having a known psychiatric pathology, being a minor, and taking treatment from the psychotropic family (e.g., neuroleptics) that could bias the results of the study due to the altered psychological state of these participants. Four groups of participants were established: no complications, GDM, risk of preterm delivery, and multiple complications.

### 2.2. Data Collection Procedure and Tools

A set of questionnaires was made available online on the Framaforms platform. The questionnaires were pilot-tested with 17 pregnant women (not included in the study) to estimate the completion time, verify question comprehension, and validate the data input. The estimated completion time was 20 min. The form consisted of a sociodemographic and obstetric questionnaire and two scales.

#### 2.2.1. Sociodemographic and Obstetric Questionnaire

This questionnaire consisted of 41 questions, 12 of which were for women with somatic pregnancy complications. It assesses sociodemographic and obstetric characteristics (current pregnancy and history). The sociodemographic information collected includes maternal age, marital status, geographical origin, employment status, educational level, support from partner, and support from family and friends. Obstetric characteristics obtained included pregnancy complications (none, risk of preterm delivery, GDM, multiple complications, and other), parity, number of miscarriages, number of abortions, number of medical terminations of pregnancy, unplanned pregnancy, difficulty getting pregnant, history of pregnancy complications, hospitalization during pregnancy, and pregnancy-related treatment. The less frequent complications (e.g., cholestasis) were categorized into an “other” group. All the obstetric data, including pregnancy complications, were self-reported.

#### 2.2.2. Edinburgh Postnatal Depression Scale (EPDS)

The EPDS [15] was used to assess depressive symptoms. This scale is a self-administered questionnaire measuring perinatal depressive symptomatology. It consists of 10 items evaluated on a 4-point Likert scale ranging from 0 (*not at all*) to 3 (*yes, most of the time*). The EPDS, initially designed to assess postnatal depression, is also widely used to assess depression during pregnancy. The French version of the EPDS validated in France [16] was used for measuring depressive symptoms. The sum of scores ranges between 0 and 30. A threshold score of 13 or more was used to identify pregnant women at high risk of depression [17].

#### 2.2.3. Maternal State and Trait Anxiety

Maternal state and trait anxiety were measured using the French version of the Spielberger State–Trait Anxiety Inventory (STAI) [18]. This is a widely used questionnaire that includes two scales to measure anxiety symptoms in clinical and nonclinical settings. The state anxiety scale assesses current symptoms of anxiety at the time of assessment, and the trait anxiety scale assesses a habitual tendency toward anxiety in general. The questionnaire contains 20 items for each anxiety scale, evaluated on a 4-point Likert scale ranging from 1 (*not at all*) to 4 (*very much*). Scores range from 20 (lowest score) to 80 (highest score). Scores equal to or above 40 indicate clinically relevant symptoms of anxiety [19, 20].

## 3. Procedure and Ethical Considerations

The study was approved by the Ethics and Protection of Personal Data Committee of Ile de France II (CPP Ile-de-France-II, N°201226, IRB 20122600001072). Participant recruitment took place online via Facebook groups (87 groups) and 17 forums such as “Future maman” or “Être enceinte.” An invitation announcement was drafted to explain the study's objective, inclusion criteria, and procedures. An information form and an informed consent form were included at the beginning of the online questionnaire. These forms informed participants about free consent, data storage modalities, the possibility of leaving the study at any time, anonymity of the data collected, and the possibility of contacting the principal investigator for any questions about the study and being referred to an appropriate care service in case of discomfort or concerns during or after participation (maternity ward psychologist or local medical–psychological center). Participants could also request the study's overall results.

## 4. Data Analysis

Descriptive statistics for sociodemographic and obstetric characteristics are presented as frequencies and percentages for categorical variables. The normality of quantitative variables was assessed using the Shapiro–Wilk test, and where the data were not normally distributed, the results were presented as medians with interquartile range (IQR). For continuous variables that followed a normal distribution, results are presented as means and standard deviation (SD). The chi-square test or Fisher's exact test was used to test the independence of categorical variables.

The median scores of EPDS and anxiety symptoms between groups with pregnancy complications and without were compared. Spearman's correlation coefficient (*r*) was used to assess the correlation between quantitative variables.

Logistic regression analyses were conducted to assess associations between mental health outcomes (depression and anxiety) and pregnancy complications using binary and continuous variables. Odds ratios and mean differences are presented as results, respectively.

Multivariable models were used to control for confounding factors while examining the association between pregnancy complications and mental health outcomes. Potential confounding factors were identified from the literature [21–29], from which we considered, for the described models, maternal age, parity, geographical origin, maternal employment status, miscarriages, planned pregnancy, marital status, maternal educational level, social support from partner, social support from family/friends, changes in lifestyle during pregnancy, and satisfaction from medical follow-up. Bivariate analyses were employed to assess the relationship of each potential confounder with the psychological variables (Table S1). A potential confounder with a *p* value of 0.1 or less than 0.1 from the bivariate analysis was kept in the adjusted models. For regression analyses and to retain good statistical power, specific types of complications were combined to indicate women with at least one complication. A *p* value of < 0.05 was considered statistically significant. Data were analyzed using STATA software (Version 16.1).

## 5. Results

A total of 492 women completed the questionnaire and were included in the present investigation. Overall, 63.0% of women presented at least one complication. The sociodemographic, psychological, and obstetric characteristics of the study participants are shown in Table 1. The proportion of women classified as at high risk of depression, elevated state anxiety, and elevated trait anxiety was 68.9%, 83.9%, and 77.4%, respectively, in the group who reported at least one pregnancy complication. The proportion of women with risk of preterm delivery, GDM, and multiple complications was 20.7%, 24.0%, and 9.8%, respectively.

Psychological outcomes according to the types of complications are presented in Table 2. In terms of anxiety scores, both trait and state anxiety scores were higher in women with multiple complications as compared to women with no complications, but statistical significance was reached only for the state anxiety scores (*p* = 0.03). EPDS scores did not differ significantly between the groups (Table 2).

Selected sociodemographic and obstetric characteristics in pregnant women with and without complications are shown in Table S1. Then, 69.81% of women with a high school diploma or less had at least one single complication as compared to 59.64% of women with a higher education diploma (*p* = 0.03). Similarly, a higher proportion of women with a history of one or more miscarriages (70.78%) presented at least one single complication as compared to women with no miscarriage history and at least one complication (59.35%; *p* = 0.02). Women who presented at least one complication during pregnancy reported significantly more often having reduced or stopped practicing a sport and a hobby (64.40%) and reduced or stopped working (82.69%) in comparison to those who had no complications (35.60% and 17.31%, respectively; *p* < 0.01). About three-quarters of women (71.43%) who did not present complications reported no change in their lifestyle during pregnancy.

Correlations between depression and anxiety symptoms in women without and with complications are presented in Tables S2 and S3, respectively. In both groups, EPDS scores were positively correlated with state and trait anxiety scores. Similarly, state and trait anxiety scores were positively correlated in women with or without pregnancy complications.

Median anxiety and depression scores by sociodemographic factors in pregnant women are shown in Table S4. State anxiety scores were significantly higher in women who felt they did not receive adequate social support during pregnancy from their partners as compared to women who perceived receiving adequate support (*p* < 0.05). State anxiety scores were also high among women who reported dissatisfaction with medical care (*p* < 0.05) and for those who reported having not received support from family and friends (*p* < 0.05). Depression and trait anxiety scores were significantly higher among women who originated from metropolitan France as compared to those who did not originate from metropolitan France (*p* < 0.05). Depression and trait anxiety scores were also significantly higher among primigravida women (*p* < 0.05).

Unadjusted and adjusted regression analyses to explore associations between pregnancy complications and psychological variables are shown in Table 3. We found positive significant associations between the presence of a pregnancy complication and high state anxiety adjusted for potential confounders (OR: 2.94; 95% CI: 1.40–6.10). When comparing psychological variables by the subtype of complication, median EPDS scores were higher in the women at risk of GDM (16.0, IQR 6 vs. 15, IQR 9, *p* = 0.018) but lower in women at risk of preterm delivery (12.5, IQR 9, *p* < 0.01). Median state anxiety scores were higher in women at risk of GDM (50, IQR 9 vs. 47, IQR 11, *p* = 0.008), while trait anxiety scores were lower in women at risk of preterm delivery (43, IQR 15, *p* < 0.01) (Table S5). After adjusting for potential confounders, significant positive associations between GDM and high risk of depressive symptoms (aOR: 1.99; 95% CI: 1.20–3.29) and high state anxiety scores (aOR: 3.31; 95% CI: 1.22–9.01) were observed (Table 4). However, there was a significant negative association between the risk of preterm delivery and high risk of depression (aOR: 0.45; 95% CI: 0.28–0.72) and elevated trait anxiety scores (aOR: 0.41; 95%: CI: 0.25–0.68).

## 6. Discussion

The present study was aimed at investigating associations between different types of pregnancy complications and psychological outcomes in a population of pregnant women in France. Our study found that, overall, 63% of our respondents had at least one pregnancy complication. Among those, there was a high proportion with a high risk of depression (68.9%), elevated state anxiety (83.9%), and high trait anxiety levels (77.4%). This corroborates existing evidence that perinatal mental health problems are high among women with high-risk pregnancies [1, 13]. Among complications, the most prevalent was GDM (24%), followed by risk of preterm delivery (20.7%) and multiple complications (9.8%).

Psychological outcomes varied according to sociodemographic factors. For instance, state anxiety scores were higher among women who did not perceive adequate social support and who reported dissatisfaction with medical care. A systematic review showed that those who lack social support tend to be exposed to loneliness, have low emotional and coping abilities, and, later, can develop anxious symptoms [12]. Furthermore, EPDS scores and trait anxiety scores were higher among women who belonged to metropolitan France and were primigravidae. However, all EPDS median scores are above the cut-off for depression among all the sociodemographic strata (Table S4). EPDS median scores are also above the cut-off in participants with pregnancy complication or without complication (Table 2). It is possible that most of our participants who may have suffered from depressive symptoms agreed to enroll themselves in the study, implying selection bias, precisely self-selection bias, possibly leading to very high EPDS scores.

 Median state anxiety scores were significantly higher among women who reported experiencing single or multiple complications as compared to women who reported experiencing no complications. Consistent with our findings, a systematic review showed elevated state anxiety among women with pregnancy complications [30].

Multivariable adjusted regression analyses showed that having a complication increases the likelihood of experiencing high state anxiety levels. Furthermore, we observed that women who suffered from GDM had a significantly higher likelihood of reporting depressive symptoms and high state anxiety scores as compared to the entire group of women without GDM. Consistent with our findings, GDM can lead to a 22%–43% increase in the likelihood of depressive disorder, according to two systematic reviews [31, 32]. Another recent meta-analysis suggested that GDM is associated with an increased risk of antenatal depression and possibly anxiety in perinatal women [9]. These studies suggest that there is some evidence of an association between GDM and mental health problems, but this relationship could be bidirectional [32]. However, the negative association between the risk of preterm delivery and the high risk of depression and elevated trait anxiety scores in this study was unexpected and inconsistent with previously conducted international large-scale studies [14, 33]. This inconsistency may be explained by the differences in the sample size, unmeasured confounders (e.g., socioeconomic status), measurement of depression, and assessment of the risk of preterm delivery in the current study. Firstly, the small sample size observed in the comparison groups is an important factor. Given the relatively small number of participants with the risk of depression and trait anxiety classified as having a high risk of preterm delivery, it is possible that we observed this statistically significant association just by chance due to random error (chance or random variation). With regard to confounders, for instance, individuals with higher SES, who are at risk of preterm delivery, may have better access to healthcare services, leading to a lower risk of depression. In terms of the measurement of depression and the risk of preterm delivery, we relied on self-reported data which could have biased the associations.

The mechanisms involved in the association between pregnancy complications and mental health problems in women are not clear; however, similar hormonal dysregulation and the same proinflammatory biomarkers such as cytokines have been detected in subjects with pregnancy-related morbidities and depression [34]. Pregnancy itself is associated with heightened emotional changes and the addition of physical morbidity during this period will increase the psychological distress of women both in the short and long term [14, 35].

This study found a high prevalence of maternal anxiety and depression in this population. Existing evidence suggests that antenatal anxiety and depression during pregnancy are associated with adverse perinatal outcomes including morbidity and mortality [36, 37]. Given the effectiveness of screening programs [38], appropriate actions should be taken to reinforce the systemic integration of mental healthcare services into the care of women suffering from pregnancy complications as recommended by national initiatives, including *Plan périnatalité*, to improve perinatal mental health [39]. It is also important to ensure the training of perinatal healthcare staff, including midwives, general practitioners, psychologists, and psychiatrists, so that they can identify and manage perinatal mental health problems and provide appropriate support to women to safeguard their physical and mental health. In France, there are guidelines to improve perinatal mental health including the ones established and regularly revised by the Haute Autorité de Santé (HAS) [40] to help healthcare professionals and social workers identify, diagnose, and manage maternal mental health problems occurring during and after pregnancy. Moreover, pathways for the management and follow-up of perinatal mental health problems have been developed, with a particular focus on the joint care of parent–baby using intersectoral approaches. However, more efforts are needed to homogenize the practices and reduce interregional disparities [40].

Another strength of this study includes collecting a large array of sociodemographic and obstetric data which was controlled in the analyses. Furthermore, using validated tools for the evaluation of depressive and anxiety symptoms in pregnant women is another strength of the study. However, our study has some limitations. Some of our study limitations include one time-point assessment of psychological variables during pregnancy and, due to the cross-sectional nature of this study, it is difficult to draw causal relationships between psychological variables and pregnancy complications. Furthermore, our sample was not fully representative of the target population, which limits the generalizability of our findings. We used a snowball sampling method potentially leading to selection bias and volunteer/nonresponse bias. The characteristics of our study sample may not be fully comparable to the target population. For example, our study included only women who had access to the internet or were users of social media (Facebook), excluding those who did not have access to the latter. Finally, the use of a self-reported questionnaire is an important limitation and may have contributed to the high rates of anxiety and depression found in this study. In addition, the use of the self-reported questionnaire to assess pregnancy complications limits the validity of the findings.

## 7. Conclusion

This study found a high prevalence of depression and anxiety among pregnant women with pregnancy complications. Overall, there was a significant association between having at least one single complication and high state anxiety levels. Furthermore, there was a significant association between GDM, antenatal depression, and high state anxiety levels. These findings highlight the need for integrating both physical and mental health healthcare services in the care of this high-risk population to ensure better health outcomes for both the expecting mothers and their children.

## Figures and Tables

**Table 1:** Sociodemographic, psychological, and obstetric characteristics in pregnant women in France (*n* = 492^a^).

		**n** ** or mean/median (SD/IQR)**	**(%)**
Sociodemographic characteristics			

Mean age (SD), years		31 (±5.01)	

Geographical origin of mother	Metropolitan France	364	73.98
	French overseas departments and territories	14	2.85
	Other	114	23.17

Geographical origin of father	Metropolitan France	343	70.43
	French overseas departments and territories	19	3.90
	Other	125	25.67

Mother's professional status	Without any professional activity	78	15.89
	Employed	413	84.11

Father's professional status	Without any professional activity	19	3.96
	Employed	461	96.04

Maternal education	No education	3	0.61
	High school diploma or less	156	31.77
	Higher education diploma	332	67.62

Marital status	Single or separated	21	4.29
	Living together	469	95.71

Psychological characteristics	Depression (≥ 13)	335	68.09
	Elevated state anxiety (≥ 40)	411	83.88
	Elevated trait anxiety (≥ 40)	381	77.44

Pregnancy complication	None	182	36.99
	Risk of preterm delivery	102	20.73
	Gestational diabetes mellitus	118	23.98
	Multiple complications	48	9.76
	Other complications	42	8.54

Parity	Primigravida	284	57.72

Number of miscarriages	≥ 1	154	31.36

Number of abortions	≥ 1	96	19.55

Number of medical terminations of pregnancy	≥ 1	26	5.30

Unintended pregnancy		95	19.31

Difficulty getting pregnant		107	21.75

History of pregnancy complications		161	32.72

Satisfaction with medical care		433	88.37

Pregnancy-related medical treatment	Yes	85	17.35

Perceived support from partner	Yes	374	83.67

Perceived support from family and friends	Yes	373	83.45

Abbreviation: IQR, interquartile range.

^a^Totals may not equal to 492 due to missing data.

**Table 2:** Median and interquartile range (IQR) of anxiety and depression according to the types of complications (no complication, single complication, and multiple complications) in pregnant women living in France (*n* = 492).

	**N**	**No complications**	**N**	**Single** ^**b**^	**n**	**Multiple**	**p** ** value** ^**a**^
EPDS	182	16 (8)	262	15 (7)	48	14.5 (6.5)	0.63
State anxiety	181	48 (9)	262	49 (11)	47	51 (13)	**0.03**
Trait anxiety	182	46 (9)	262	46 (12)	48	47 (12)	0.95

*Note:* Bold number means that the result is statistically significant.

^a^Kruskal–Wallis test.

^b^Preterm delivery, gestational diabetes mellitus, and others were combined to form the category of “single complication.”

**Table 3:** Multiple logistic regression of anxiety and depression scores according to the status of complications in pregnant women living in France (*n* = 492).

	**High risk of depression**			**Elevated state anxiety**			**Elevated trait anxiety**		
	**n** ** (%)**	**Crude OR (95% CI)**	**Adjusted OR** ^**a**^ ** (95% CI)**	**n** ** (%)**	**Crude OR (95% CI)**	**Adjusted OR (95% CI)** ^**b**^	**n** ** (%)**	**Crude OR (95% CI)**	**Adjusted OR (95% CI)** ^**c**^
Pregnancy complication									
Absent	1130 (38.8)	Reference	Reference	147 (35.8)	Reference	Reference	151 (39.6)	Reference	Reference
Present	2205 (61.2)	0.78 (0.52–1.16)	0.85 (0.56–1.28)	264 (64.2)	1.36 (0.83–2.21)	**2.94 (1.40–6.14)**	230 (60.4)	0.59 (0.37–0.94)	0.65 (0.40–1.05)

*Note:* Cells with significant OR are presented in bold.

^a^Adjusted for primigravida, geographical origin of mother, and unplanned pregnancy.

^b^Adjusted for social support from partner, social support from family and friends, primigravida, maternal age, maternal depression, and geographical origin of mother.

^c^Adjusted for maternal age, marital status, maternal educational level, unplanned pregnancy, social support from husband/partner, social support from family and friends, maternal depression, primigravida, and geographical origin of mother.

**Table 4:** Multiple logistic regression of anxiety and depression scores according to the complication subtype in pregnant women living in France (*n* = 492).

	**High risk of depression**			**Elevated state anxiety**			**Elevated trait anxiety**		
	**n** ** (%)**	**Crude OR (95% CI)**	**Adjusted OR** ^**a**^ ** (95% CI)**	**n** ** (%)**	**Crude OR (95% CI)**	**Adjusted OR (95% CI)** ^**b**^	**n** ** (%)**	**Crude OR (95% CI)**	**Adjusted OR (95% CI)** ^**c**^
Gestational diabetes									
Absent	209 (69.4)	Reference	Reference	261 (70.9)	Reference	Reference	247 (71.6)	Reference	Reference
Present	92 (30.6)	1.91 (1.18–3.10)	**1.99 (1.20–3.29)**	107 (29.1)	2.39 (1.2–4.7)	**3.31 (1.22–9.01)**	98 (28.4)	1.57 (0.91–2.70)	1.56 (0.89–2.73)
Risk of preterm delivery									
Absent	250 (83.1)	Reference	Reference	290 (78.80)	Reference	Reference	283 (82.0)	Reference	Reference
Present	51 (16.94)	0.40 (0.23–0.58)	**0.45 (0.28–0.72)**	78 (21.20)	0.57 (0.33–0.99)	0.88 (0.41–1.89)	62 (17.8)	0.32 (0.20–0.53)	**0.41 (0.25–0.68)**

*Note:* Cells with significant OR are presented in bold.

^a^Adjusted for primigravida, geographical origin of mother, and unplanned pregnancy.

^b^Adjusted for social support from husband/partner, social support from family and friends, primigravida, maternal age, maternal depression, and geographical origin of mother.

^c^Adjusted for maternal age, marital status, maternal educational level, unplanned pregnancy, social support from husband/partner, social support from family and friends, maternal depression, primigravida, and geographical origin of mother.

## Data Availability

The data that support the findings of this study are available from the corresponding author upon reasonable request.

## Supporting Information

Supporting Information Additional supporting information can be found online in the Supporting Information section. Supporting information tables are available: Table S1: Selected sociodemographic and obstetric characteristics in pregnant women by the presence of complications. Table S2: Correlations between depression and anxiety symptoms in pregnant women without complications. Table S3: Correlations between depression and anxiety symptoms in pregnant women with single or multiple complications. Table S4: Median anxiety and depression scores by selected sociodemographic factors in pregnant women. Table S5: Median and interquartile range (IQR) of anxiety and depression scores according to the gestational diabetes mellitus (GDM) status in pregnant women.
