# Supplementary material for: High Risk of Anxiety and Depression in Women With Different Types of Pregnancy Complications in France: A Cross-Sectional Study
Source: J Pregnancy. 2025 Dec 1;2025:9221290. doi: 10.1155/jp/9221290 (PMC12685426; doi:10.1155/jp/9221290)
Supplement: Supporting Information — Additional supporting information can be found online in the Supporting Information section. Supporting information tables are available: Table S1: Selected sociodemographic and obstetric characteristics in pregnant women by the presence of complications. Table S2: Correlations between depression and anxiety symptoms in pregnant women without complications. Table S3: Correlations between depression and anxiety symptoms in pregnant women with single or multiple complications. Table S4: Median anxiety and depression scores by selected sociodemographic factors in pregnant women. Table S5: Median and interquartile range (IQR) of anxiety and depression scores according to the gestational diabetes mellitus (GDM) status in pregnant women. [file 9221290.f1.zip › Supplementary Tables.docx]

**Supplementary** **Table 1. Selected sociodemographic and obstetrical characteristics in pregnant women by the presence of complication (n=492*).**

|  | No complication | At least one complication |  |
| --- | --- | --- | --- |
|  | n (%) | n (%) | P |
| Sociodemographic factors |  |  |  |
| Marital status |  |  |  |
| Single or separated | 5 (23.81) | 16 (76.19) | 0.20 |
| Living in couple | 177 (37.74) | 292 (62.26) |  |
| Social support from partner |  |  |  |
| Yes | 145 (38.77) | 229 (61.23) | 0.61 |
| Social support from family/ friends |  |  |  |
| Yes | 138 (37.00) | 235 (63.00) | 0.22 |
|  |  |  |  |
| Geographical origin of mother |  |  |  |
| Metropolitan France | 141 (38.74) | 223 (61.26) | 0.18 |
| Others | 41 (32.03) | 87 (67.97) |  |
| Educational level of mother |  |  |  |
| High school diploma or less | 48 (30.19) | 111 (69.81) | **0.03** |
| Higher education diploma | 134 (40.36) | 198 (59.64) |  |
| Maternal age |  |  |  |
| <30 | 67 (36.22) | 118 (63.78) | 0.78 |
| >30 | 115 (37.46) | 192 (62.54) |  |
| Maternal professional status |  |  |  |
| Unemployed | 28 (35.90) | 50 (64.10) | 0.82 |
| Employed | 154 (37.29) | 259 (62.71) |  |
|  |  |  |  |
| Primigravida | 114 (40.14) | 170 (59.86) | 0.09 |
|  |  |  |  |
| Number of miscarriages |  |  |  |
| None | 137 (40.65) | 200 (59.35) | **0.02** |
| 1 or more | 45 (29.22) | 109 (70.78) |  |
| Number of abortions |  |  |  |
| >1 | 34 (35.42) | 62 (64.58) |  |
| Planned pregnancy |  |  |  |
| Yes | 147 (37.03) | 250 (62.97) | 0.97 |
|  |  |  |  |
| Hospitalisation |  |  |  |
| Yes | 0 (0.0) | 149 (100.0) | 0.34 |
| Satisfaction with medical care |  |  |  |
| Yes | 162 (37.41) | 271 (62.59) | 0.73 |
|  |  |  |  |
| Changes in lifestyle during pregnancy |  |  |  |
| None | 50 (71.43) | 20 (28.57) | **<0.01** |
| Reduction or cessation sport/ hobby | 110 (35.60) | 199 (64.40) |  |
| Reducing or stopping work | 9 (17.31) | 43 (82.69) |  |

*Totals may not equal to 492 due to missing data.

**Supplementary** **Table 2: Correlations between depression and anxiety symptoms in pregnant women without complications (n=181).**

|  | EPDS | State-Anxiety | Trait-Anxiety |
| --- | --- | --- | --- |
| EPDS | 1.00 |  |  |
| State-Anxiety | 0.33* | 1.00 |  |
| Trait-Anxiety | 0.39* | 0.42* | 1.00 |
|  |  |  |  |

p <0.05: *

**Supplementary** **Table 3: Correlations between depression and anxiety symptoms in pregnant women with single or multiple complications (n=309).**

|  | EPDS | State-Anxiety | Trait-Anxiety |
| --- | --- | --- | --- |
| EPDS | 1.00 |  |  |
| State-Anxiety | 0.37* | 1.00 |  |
| Trait-Anxiety | 0.46* | 0.46* | 1.00 |
|  |  |  |  |

p <0.05: *

**Supplementary** **Table 4. Median anxiety and depression scores by selected sociodemographic factors in pregnant women (n=492).**

| Sociodemographic factors | EPDS | State-Anxiety | Trait-Anxiety |
| --- | --- | --- | --- |
| Marital status |  |  |  |
| Living in couple | 15 | 48 | 46 |
| Separated | 15 | 47 | 47 |
| Social support from partner |  |  |  |
| Yes | 16 | 49** | 47 |
| No | 15 | 51 | 47 |
| Social support from family and friends |  |  |  |
| Yes | 16 | 49 ** | 47 |
| No | 15.5 | 51 | 46 |
| Geographical origin of mother |  |  |  |
| Metropolitan France | 16** | 49* | 47** |
| Others | 13 | 47 | 43 |
| Educational level of mother |  |  |  |
| High school diploma or less | 15 | 49 | 46 |
| Higher education diploma | 16 | 48 | 47 |
| Maternal age |  |  |  |
| <30 | 16 | 49* | 46 |
| >30 | 15 | 48 | 46 |
| Maternal Professional status |  |  |  |
| Unemployed | 15 | 46 | 44 |
| Employed | 15 | 49 | 46 |
| Parity |  |  |  |
| Primigravida | 16** | 49* | 45** |
| No | 15 | 47.5 | 47 |
| Number of abortions |  |  |  |
| 0 | 15 | 48 | 46 |
| >1 | 16 | 49 | 47 |
| Planned pregnancy |  |  |  |
| Yes | 16* | 48 | 46 |
| No | 14 | 48 | 47 |
| Satisfaction with medical care |  |  |  |
| Yes | 15 | 48** | 46 |
| No | 16 | 52 | 46 |
| Change in lifestyle after |  |  |  |
| None | 16 | 49 | 47 |
| Reduction or cessation of sport or a hobby | 16 | 49 | 47 |
| Reducing or stopping of work | 14.5 | 49.5 | 48 |

p <0.05: **

p <0.1: *

**Supplementary Table 5. Median and interquartile range (IQR) of anxiety and depression scores according to the Gestational Diabetes Mellitus (GDM) status in pregnant women (n=492).**

|  |  | GDM | | |  | risk of preterm delivery | | | | |  | |
| --- | --- | --- | --- | --- | --- | --- | --- | --- | --- | --- | --- | --- |
|  | n | Absent | N | Present | *P* | n | Absent | n | Present | *p* | |  |
| EPDS | 326 | 15 (9) | 118 | 16 (6) | **0.018** | 342 | 16 (7) | 102 | 12.5 (9) | **0.005** | |  |
| State-Anxiety | 325 | 47 (11) | 118 | 50 (9) | **0.008** | 341 | 49 (9) | 102 | 45.5 (14) | **0.12** | |  |
| Trait-Anxiety | 326 | 46 (10) | 118 | 48 (9) | **0.011** | 342 | 47 (9) | 102 | 43 (15) | **0.01** | |  |

GDM: Gestational Diabetes Mellitus
