# Supplementary material for: High Risk of Anxiety and Depression in Women With Different Types of Pregnancy Complications in France: A Cross-Sectional Study
Source: J Pregnancy. 2025 Dec 1;2025:9221290. doi: 10.1155/jp/9221290 (PMC12685426; doi:10.1155/jp/9221290)

Questionnaire réalisé sur Framaform

#### Questionnaire de la grossesse : 2 / 9

Quel est votre âge ? *

Quelle est votre origine ? *

 France métropolitaine

 DOM-TOM

 Autre

Choisir une seule réponse parmi celles ci-dessus.

Si Autre, précisez *

Quelle est l'origine du père de votre bébé ? *

 France métropolitaine

 DOM-TOM

 Autre

 Pas en contact avec le père - je ne sais pas

Choisir une seule réponse parmi celles ci-dessus.

Si Autre, précisez *

Quelle est votre profession ? *

Quelle est votre situation professionnelle actuelle ? *

 Travail à temps plein

 Travail intérimaire ou intermittent

 Travail à temps partiel

 Congé parental

 Etudiant

 Chômage

 Au foyer

 Autre

Choisir une seule réponse parmi celles ci-dessus.

Si Autre, précisez *

Quelle est la profession du père de votre bébé ? *

Quelle est votre situation conjugale ? *

 Célibataire

 En couple (concubinage, mariée, pacsée)

 Divorcée/séparée

 Autre

Choisir une seule réponse parmi celles ci-dessus.

Si Autre, précisez *

Cochez le niveau d’études le plus élevé que vous avez atteint *

 Aucun diplôme

 Collège sans BEPC, CAP ou BEP

 BEPC, CAP ou BEP

 Lycée sans BAC

 BAC ou Brevet professionnel

 Diplômes supérieurs

Cochez le niveau d’études le plus élevé atteint par votre conjoint *

 Aucun diplôme

 Collège sans BEPC, CAP ou BEP

 BEPC, CAP ou BEP

 Lycée sans BAC

 BAC ou Brevet professionnel

 Diplômes supérieurs

 Pas en contact avec le père - je ne sais pas

Depuis combien de temps êtes-vous enceinte ? (en semaine) *

Combien avez-vous d’enfants ? *

Aviez-vous déjà été enceinte ? *

 Oui

 Non

(Y compris d’éventuelles grossesses non menées à terme)

Avez-vous fait des fausses couches ? *

 Oui

 Non

Avez-vous fait d’Interruptions Volontaires de Grossesse (IVG) ? *

 Oui

 Non

Avez-vous fait d’Interruptions Médicales de Grossesse (IMG) ? *

 Oui

 Non

Cette grossesse était-elle prévue ? *

 Oui

 Non

Avez-vous eu des difficultés à tomber enceinte pour cette grossesse ? *

 Oui

 Non

Avez-vous suivi un traitement particulier ? *

 Oui

 Non

Si oui, lequel ? *

#### Questionnaire de la grossesse : 3 / 9

Avez-vous rencontré des complications au cours des grossesses précédentes ? *

 Oui

 Non

 Je vis ma première grossesse

Si oui, précisez *

Avez-vous dû changer votre mode de vie pendant votre grossesse actuelle ? *

 Non

 Diminution ou arrêt du sport

 Diminution ou arrêt d'un loisir

 Réduction ou arrêt de votre travail

 Changement dans votre alimentation (ex. régime hypocalorique, hyposodé)

 Autre

Si Autre *

Avez-vous rencontré ou rencontrez-vous des complications lors de votre grossesse actuelle ? *

 Oui

 Non

Si oui, précisez *

 Saignements vaginaux

 Hypertension

 Menace d'accouchement prématuré

 Diabète gestationnel

 Autre

Si Autre *

A quel(s) moment(s) de votre grossesse? (en semaines de grossesse) *

Avez-vous été arrêtée par un médecin pendant votre grossesse actuelle? *

 Oui

 Non

A quel moment de votre grossesse avez-vous été arrêté la première fois ? (en semaines de grossesse) *

En cas de deuxième arrêt, à quel moment de votre grossesse l'avez-vous été ? (en semaines de grossesse)

Combien de temps avez-vous été arrêtée la première fois ? (en nombre de jours) *

Combien de temps avez-vous été arrêtée la deuxième fois ? (en nombre de jours)

Avez-vous été hospitalisée en raison de ces complications? *

 Oui

 Non

Si oui, pendant combien de temps la première fois ? (en nombre de jours) *

Si vous avez été hospitalisée une deuxième fois, pendant combien de temps ? (en nombre de jours)

Jugez-vous que ces complications ont ou ont eu un impact sur votre vie quotidienne (qualité de vie): *

 Impact très négatif

 Impact négatif

 Impact positif

 Aucun impact

Pourquoi ? *

En référence à la question précédente

Pensez-vous que ces complications ont un impact sur votre relation avec votre futur enfant ? *

 Oui

 Non

Précisez la question précédente *

Jugez-vous que le suivi médical de ces complications est-il: *

 Très contraignant

 Plus ou moins contraignant

 Pas contraignant

Etes-vous satisfaite du suivi médical qui vous est proposé ? *

 Oui

 Non

Précisez *

En référence à la question précédente

Un accompagnement psychologique vous a-t-il été proposé pendant votre grossesse? *

 Oui

 Non

Si oui, l’avez-vous accepté ? *

 Oui

 Non

Pour quelles raisons ? *

Etes-vous suivie actuellement par un psychologue ou psychiatre ? *

 Oui

 Non

Avez-vous actuellement un traitement médicamenteux psychologique ? *

 Oui (Antidépresseur, Thymorégulateur, Anxiolytique, Somnifère, Neuroleptique, Antipsychotique)

 Non

Précisez le(s) nom(s): *

*Vous sentez-vous suffisamment soutenue pendant votre grossesse*

par le père de l'enfant? *

 Oui

 Non

par votre entourage proche (famille et/ou amis)? *

 Oui

 Non

Avez-vous des inquiétudes au sujet de votre grossesse et/ou de votre futur enfant? *

#### EPDS –

#### Questionnaire 2 : 5 / 9

Vous allez avoir un bébé. Nous aimerions savoir comment vous vous sentez. Nous vous demandons de bien vouloir remplir ce questionnaire en soulignant la réponse qui vous semble le mieux décrire comment vous vous êtes sentie durant la semaine (c’est-à-dire dans les 7 jours qui viennent de s’écouler) et pas seulement aujourd’hui.

Voici un exemple : Je me suis sentie heureuse :
o Oui, tout le temps
• Oui, la plupart du temps
o Non, pas très souvent
o Non, pas du tout
Cela signifiera « Je me suis sentie heureuse la plupart du temps durant la semaine qui vient de s’écouler ».

Merci de bien vouloir répondre aux autres questions.

Pendant la semaine qui vient de s’écouler :

1. J’ai pu rire et prendre les choses du bon côté. *

 Aussi souvent que d'habitude

 Pas tout à fait autant

 Vraiment beaucoup moins souvent ces jours-ci

 Absolument pas

2. Je me suis sentie confiante et joyeuse, en pensant à l’avenir. *

 Autant que d’habitude

 Plutôt moins que d’habitude

 Vraiment moins que d’habitude

 Pratiquement pas

3. Je me suis reprochée, sans raisons, d’être responsable quand les choses allaient mal. *

 Oui, la plupart du temps

 Oui, parfois

 Pas très souvent

 Non, jamais

4. Je me suis sentie inquiète ou soucieuse sans motifs. *

 Non, pas du tout

 Presque jamais

 Oui, parfois

 Oui, très souvent

5. Je me suis sentie effrayée ou paniquée sans vraiment de raisons. *

 Oui, vraiment souvent

 Oui, parfois

 Non, pas très souvent

 Non, pas du tout

6. J’ai eu tendance à me sentir dépassée par les événements. *

 Oui, la plupart du temps, je me suis sentie incapable de faire face aux situations

 Oui, parfois, je ne me suis pas sentie aussi capable de faire face que d’habitude

 Non, j’ai pu faire face à la plupart des situations

 Non, je me suis sentie aussi efficace que d’habitude

7. Je me suis sentie si malheureuse que j’ai eu des problèmes de sommeil. *

 Oui, la plupart du temps

 Oui, parfois

 Pas très souvent

 Non, jamais

8. Je me suis sentie triste ou peu heureuse. *

 Oui, la plupart du temps

 Oui, très souvent

 Pas très souvent

 Non, pas du tout

9. Je me suis sentie si malheureuse que j’en ai pleuré. *

 Oui, la plupart du temps

 Oui, très souvent

 Seulement de temps en temps

 Non, jamais

10. Il m’est arrivé de penser à me faire du mal. *

 Oui, très souvent

 Parfois

 Presque jamais

 Jamais

STAI – état

Questionnaire 3 : 6 / 9


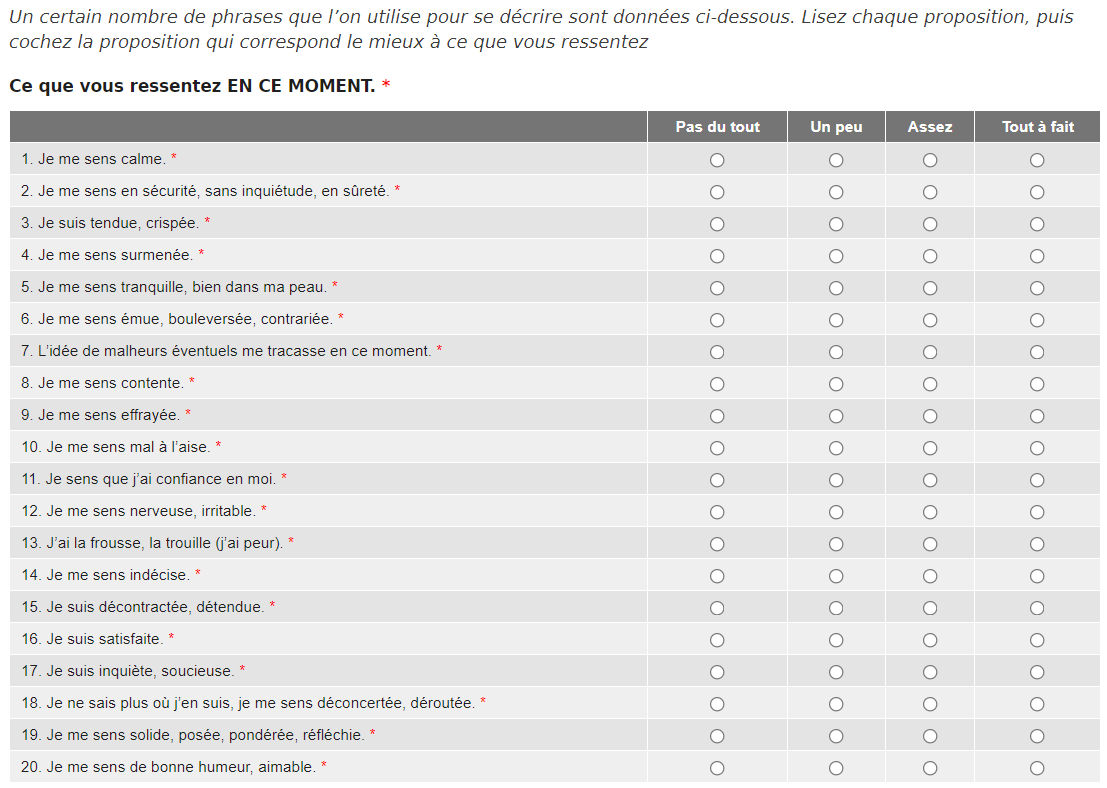


STAI – trait


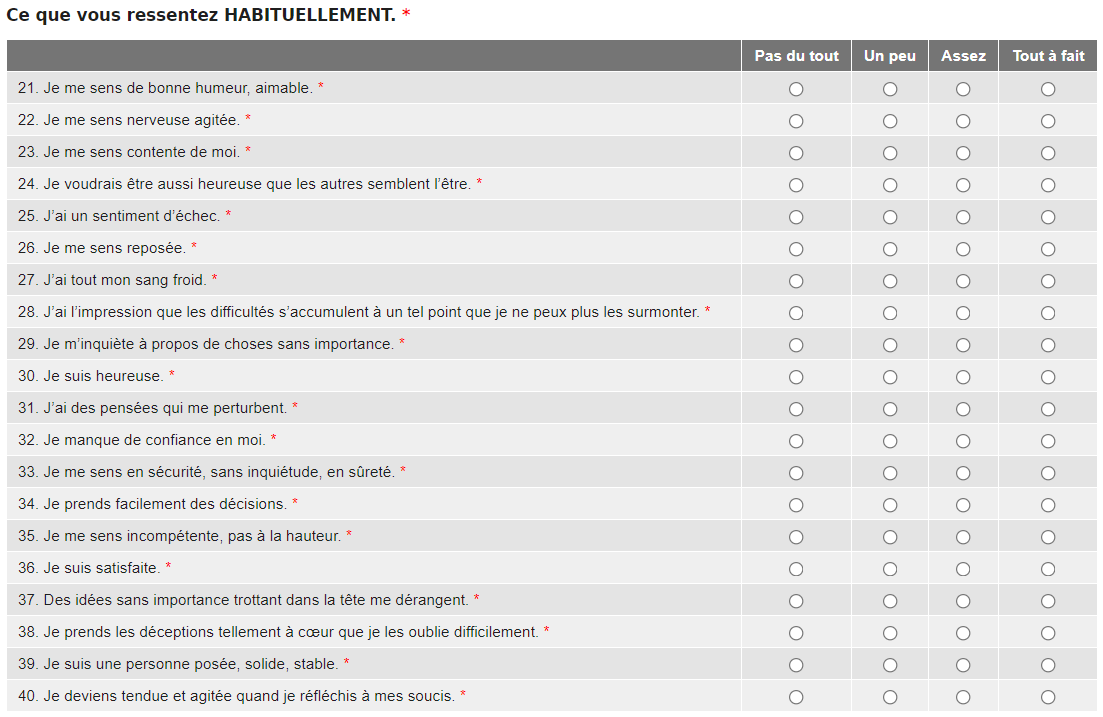

Supplement: Supporting Information — Additional supporting information can be found online in the Supporting Information section. Supporting information tables are available: Table S1: Selected sociodemographic and obstetric characteristics in pregnant women by the presence of complications. Table S2: Correlations between depression and anxiety symptoms in pregnant women without complications. Table S3: Correlations between depression and anxiety symptoms in pregnant women with single or multiple complications. Table S4: Median anxiety and depression scores by selected sociodemographic factors in pregnant women. Table S5: Median and interquartile range (IQR) of anxiety and depression scores according to the gestational diabetes mellitus (GDM) status in pregnant women. [file 9221290.f1.zip › supplementary material_questionnaires.docx]
